# Susceptibility to Coronavirus (COVID-19) in Occupational Settings: The Complex Interplay between Individual and Workplace Factors

**DOI:** 10.3390/ijerph18031030

**Published:** 2021-01-25

**Authors:** Veruscka Leso, Luca Fontana, Ivo Iavicoli

**Affiliations:** Department of Public Health, Section of Occupational Medicine, University of Naples Federico II, Via S. Pansini 5, 80131 Naples, Italy; veruscka.leso@unina.it (V.L.); luca.fontana@unina.it (L.F.)

**Keywords:** SARS-CoV-2, vulnerability, frailty, risk factors, severe COVID-19, mortality, comorbidities, risk assessment, risk management, occupational health

## Abstract

In the current coronavirus (COVID-19) pandemic, the definition of risk factors for susceptibility to adverse outcomes seems essential to support public and occupational health policies. Some specific issues need to be addressed to understand vulnerability in occupational settings. Among these, individual factors, e.g., age, sex, and preexisting comorbidities (hypertension, cardiovascular diseases, diabetes, obesity, cancer), that can predispose individuals to more severe outcomes and post-COVID-19 symptoms that may represent conditions of acquired susceptibility, possibly impacting the return to—and fitness for—work. Additionally, the risk of contracting COVID-19 through work should be addressed, considering the probability of being in contact with infected people, physical proximity to others, and social aggregation during work. Occupational health settings may represent appropriate scenarios for the early identification of vulnerable subjects, with the final aim to guide risk assessment and management procedures. These should include the systematic surveillance of work-related risk factors, collective preventive policies, stringent actions for specific groups of workers, decisions on occupational placement of employees, and health promotion activities. Concerted actions of general practitioners, hospital specialists, occupational physicians, and all the stakeholders involved in the occupational health and safety management should be focused on planning suitable preventive measures for susceptible subjects.

## 1. Introduction

In the current coronavirus (COVID-19) pandemic, the definition of risk factors for susceptibility to adverse and mortality outcomes represents an essential tool to support disease management issues. This can be useful to better define the clinical and epidemiological characteristics of the disease and to facilitate decision-making regarding the appropriate care for patients. From a preventive perspective, considering that countries are adapting to the longer-term challenges of COVID-19, a deeper knowledge of suscetibility risk factors may provide guidance for the development of suitable policies to protect the public and occupational health [1].

To this aim, it is important to consider the huge toll paid by frail people in terms of severe clinical manifestations, access to intensive care units and mortality, in the early stage of the COVID-19 pandemic. Although frailty has its own clinical definition, as it is intended as “a medical syndrome with multiple causes and contributors, that is characterized by diminished strength, endurance and reduced physiologic function that increases an individual’s vulnerability for developing increased dependency and/or death” [2,3], data extrapolated from the experience of frail patients may be useful to identify conditions of susceptibility requiring specific preventive actions to possibly avoid the infection and achieve better management of the disease.

Since its first recognition in China in December 2019, the disease has spread around the globe at an unprecedented pace. At the time this review was written, more than 76 million confirmed cases of COVID-19, including more than 1,700,000 deaths, have been reported worldwide [4]. The US has experienced more deaths from COVID-19 than any other country and has one of the highest cumulative per capita death rates [5]. Italy has been one of the first and most severely affected countries [6], with 1,977,370 confirmed cases of COVID-19 and 69,842 deaths from 3 January to 23 December 2020 [7].

Additionally, worldwide reports have confirmed the high risk of severe illness from COVID-19, including the need for hospitalization, intensive care and death, in older adults, particularly in those with comorbidities (e.g., cardiovascular and respiratory diseases, morbid obesity, diabetes, chronic kidney disease, cancer, gastrointestinal, skin, muscle-skeletal, and immune diseases) [8,9,10,11,12,13].

Acquiring this kind of information seems critically important for occupational physicians who are increasingly asked to advise on the fitness for work of patients who may be unusually vulnerable to the disease. It is essential to support employers in the adoption of suitable preventive and protective measures, including collective, individual, or organizational interventions, aimed to protect the health of workers, particularly for those at higher risk of unfavorable outcomes. In this scenario, additional efforts for occupational physicians are required to define the complex interplay between susceptibility conditions to COVID-19 with respect to specific occupational contexts and risk factors. Therefore, the aim of this narrative review is to provide an updated overview on possible individual risk factors predictive for unfavorable mortality and morbidity outcomes that may inform risk assessment and management in workplace scenarios, with the perspective to identify knowledge gaps and future research needs in this field.

## 2. Literature Search

Advanced searches on PubMed, Scopus, and ISI Web of Science databases were conducted to identify studies published until the 20 December 2020, evaluating possible physiological or pathological conditions of susceptibility to SARS-CoV-2 infection and adverse outcomes, as well as relevant issues to be considered for the consequent risk assessment and management processes in different occupational sectors, taking into particular account the protection of vulnerable workers.

We used two search lines that included the terms “COVID-19” or “SARS-CoV-2”, combined with “frailty” or “susceptibility” or “vulnerability”, which were further combined with additional individual terms referring to possible conditions influencing disease manifestation and course: “age”, “gender”, “cardiovascular disease*”, “diabetes”, “overweight and obesity”, “cancer”, “respiratory disease*”, “autoimmune disease*”. After removal of duplicates, the titles and abstracts of the articles retrieved through the computerized search were independently examined by two of the authors. All types of human peer-reviewed research articles (i.e., descriptive epidemiological surveys, cross-sectional, cohort, case-control studies, case series), as well as review papers, were included. Only studies published in languages other than English and publications not specifically focusing on the topics of the review were excluded. For the section on occupational risk factors for susceptibility to COVID-19, the first two search lines were combined with the terms “occupational risk assessment” and “occupational risk management”. Furthermore, some additional documents belonging to the gray literature from national and international governmental and research institutes involved in occupational health and safety management were considered for inclusion.

As the review process was not intended to be systematic, and the idea of the authors was to provide inputs on relevant topics in the occupational health practice, articles considered better representative of relevant issues in the respective fields were discussed among the authors and finally included in such a critical revision. The following paragraphs will attempt to summarize important aspects regarding individual and occupational risk factors for COVID-19 that should be carefully considered for adequate protection of the health of workers, particularly for those with conditions of greater susceptibility to acquire and have a more severe course of the disease.

## 3. Individual Risk Factors for SARS-CoV-2 Susceptibility

### 3.1. Age

A great series of epidemiological and observational studies demonstrated an increased number of cases and a greater risk of severe and fatal disease with increasing age [14,15,16,17,18,19]. Older peoples’ immune-senescence may be characterized by the disruption in both innate and adaptive arms of the immune system, which is not efficient enough to limit infection, as demonstrated by the higher peak viral load in the nasopharynx determined in older adults [20,21]. This results in an increased vulnerability and poor resolution of homeostasis following stress, which may lead to low resilience and frailty. In addition, it seems that very high pro-inflammatory cytokine release, which is described as a cytokine storm, is a pivotal pathophysiological mechanism in elderly COVID-19 patients [22]. Although the exact underlying mechanism of cytokine storm in elderly adults with severe COVID-19 infection is far from clear, it is likely that such dysregulation of the cytokine homeostasis may play a critical role in the development of the acute respiratory distress syndrome (ARDS) in some elderly patients. Many age-related pathophysiologic processes have been suggested as potentially related to such “inflame-aging phenomenon”, among those, the alteration of the expression of the angiotensin-converting enzyme 2 (ACE2) receptor [23], the excessive reactive oxygen species production [24], the autophagy alterations [25], the inflammatory phenotype of senescent cell activity, particularly adipose tissue [26], and immune-senescence [27], as well as lack of vitamin D content [28]. Additionally, aging-related worst outcomes may be dependent on the intrinsic pathophysiological changes that characterize the respiratory system, as well as by the increasing presence of chronic comorbidities with increasing age [29]. However, attention should be paid to consider chronological age per se without considering that individuals do not age the same and that the underlying mechanisms of aging may be different between subjects.

### 3.2. Gender

First reports from China have pointed out a sex imbalance with regard to detected cases and case fatality rate of COVID-19 [30,31,32,33]. The largest body of publicly available sex-disaggregated data demonstrated no gender-related differences in the number of confirmed cases, with 45.7% female vs. 54.3% male cases in September 2020 [34,35], in line with previously published data [36,37]. However, the overall case-fatality ratio was approximately 2.4 times higher among men than among women [38,39]. Clinical and epidemiological evidence from China [37], the US [40], the UK [41] and European countries (Italy, Spain, France, Germany and Switzerland) [36] demonstrated a 60% to 80% increase in the risk of death for males compared to females. The diverse regulation of the immune system determined both by sex chromosome genes, and sex hormones can be responsible for gender-related differences in COVID-19 mortality [38,42,43]. Moreover, the higher tissue expression of the ACE2 receptors for SARS-CoV-2 in men can cause a greater susceptibility to the infection [44,45]. Psychosocial and behavioral factors can also play a role [46,47,48]. In fact, due to a different belief of the pandemic severity, men tend to engage in more high-risk behaviors, have lower rates of social distancing, wearing masks, and seeking medical help compared to women, thus enhancing the possibility for SARS-CoV-2 infection [49,50,51]. Smoking and drinking rates are higher among men than women, and such behaviors may be associated with the risk of developing comorbidities [36]. Possible differences in occupational risks between men and women may account for diverse COVID-19 outcomes. In the US, women are primarily employed in social and healthcare activities, while men are mainly involved in low-skilled or low-paid essential occupations, e.g., food processing, transportation, delivery, warehousing, construction, manufacturing, where men may experience a greater risk of mortality [52].

### 3.3. Cardiovascular Risk Factors

Hypertension has been reported as the most common comorbidity among patients infected with SARS-CoV-2, with a prevalence ranging from 10–15% to more than 50% [53,54,55,56,57,58]. Hypertension has been proven to be associated with an increased risk of pneumonia, as well as acute respiratory disease and chronic low respiratory disorders independent of age, sex, smoking status and BMI [59,60]. The prevalence of hypertension was higher in patients with severe COVID-19 [61,62,63,64] and with lethal outcomes [65,66,67,68] with respect to non-severe cases. However, to achieve a correct interpretation of these data, it should be noted that hypertension is commonly diagnosed in aged populations, and it is accompanied by many comorbidities, i.e., obesity, diabetes, and cardiovascular diseases (CVDs), that may function as major determinants for COVID-19 severity. Concerning age, hypertension was reported in percentages ranging from 44.7% of the 55–64 years old Chinese population [69], in the 45.2% of the Italian population aged 60–69 years [70], up to the 63–77% of the US 55–64 years general population [71]. Overall, this underlines the relevance to further understand whether hypertension was a predictor of mortality independently of other concomitant risk factors [63].

Additionally, also CVDs, i.e., heart failure and arrhythmia, appear as risk factors for severe complications of COVID-19 [53]. Patients with a previous history of CVDs were up to 4 and 6 times more likely to develop the severe and lethal disease, respectively [72]. In line with these findings, data from the Chinese Center for Disease Control and Prevention pointed to a 10.5% mortality rate in COVID-19 patients with CVD compared to a lower 2.3% rate in the overall infected population [73]. Moula et al. [74], in a meta-analysis, assessed the predictive role of different CVDs and coronary artery disease (CAD) separately with respect to COVID-19 mortality. The presence of other CVDs is a strong predictor of death. In fact, a 1.96- and 1.90-fold higher mortality risk was reported in patients with overall CVD and CAD alone, respectively, while these 2 conditions combined were found to increase the risk up to 2.03-fold. Cerebrovascular disease is a strong predictor of death as patients with the preexisting cerebrovascular disease tend to die 2-fold more than patients without cerebrovascular disease [74,75]. In addition to a history of CVD, being an aggravating factor in COVID-19 development, cardiovascular complications can also be the result of the infection, both through a direct SARS-CoV-2 induced myocardial injury as well as by an indirect action exerted by the acute respiratory distress syndrome and systemic illness [76,77,78].

### 3.4. Diabetes

Most of the available information refers to patients with type 2 diabetes. This disease per se does not seem to increase the risk of contracting COVID-19 [79]. The prevalence of type 2 diabetes in patients with COVID-19 has been reported to be around 9–12% [80,81,82], raising up to 16–35% in hospitalized patients, including those who required intensive care for the severe disease [83,84,85]. Type 2 diabetes, in fact, has been reported to be more often associated with severe or critical disease, including ARDS, multiorgan failure and death, varying from 14 to 32% in different studies [59,86,87,88]. Additionally, in a summary report of 44,672 confirmed COVID-19 cases, the Chinese Center for Disease Control and Prevention reported an increased case fatality rate in diabetes (2.3% overall and 7.3% in diabetic patients) [89].

As regards different types of diabetes, a UK survey found that among 23,804 COVID-19 patients died in hospital, 32% had type 2 diabetes, and 1.5% had type 1 diabetes, with 2.03 and 3.5 times the odds of dying compared with patients without diabetes, respectively. A multicenter observational French study, that was performed on 1317 diabetic patients hospitalized for COVID-19, showed that 3% had type 1 diabetes, while 88.5% were affected by type 2 disease [90].

Concerning the impact of glycemic control on COVID-19 mortality, patients with an HbA1c of 86 mmol/mol (10.0%) or higher had increased COVID-19-related mortality compared to people with an HbA1c of 48–53 mmol/mol (6.5–7.0%), with a hazard ratio of 2.23 and 1.61 in type 1 and type 2 diabetes, respectively. In addition, in people with type 2 diabetes, those with an HbA1c of 59–74 mmol/mol (7.6–8.9%) and 75–85 mmol/mol (9.0–9.9%) showed a significantly higher COVID-19 mortality compared with those with an HbA1c of 48–53 mmol/mol, with an HR of 1.22 and 1.36, respectively [91].

In summary, a worse prognosis is expected in patients with diabetes affected by COVID-19, most probably because of the concurring effect of multiple factors, i.e., age, comorbidities (hypertension, CVDs and obesity), and glycemic control before-, at the time of- and during hospital admission [79]. Many uncertainties still remain regarding the mechanisms linking diabetes with a more severe course of the disease. The high expression of ACE2 receptors used by SARS-CoV-2 to enter human cells, poor glucose control and high glycemic variability, preexisting diabetes-induced target organ damage, increased inflammatory factors, and hyper-coagulability can be responsible for higher susceptibility of diabetic patients to the adverse outcomes of the infection [82,92].

### 3.5. Overweight and Obesity

Emerging evidence supports excessive body weight and obesity as risk factors for more serious COVID-19 disease and fatal outcomes [93,94,95,96]. A retrospective analysis of the body mass index (BMI) in USA SARS-CoV-2 infected patients demonstrated that subjects aged <60 years with a BMI between 30 and 34 and >35 were about two and four times more likely to be admitted to acute and critical care compared to individuals with a BMI < 30 [97], respectively. Other case studies confirmed obesity as a major risk factor for disease severity and intensive care unit requirements both in the US [53,83], China [98], the UK [99], France [100,101], Germany [102], and Italy [103,104]. COVID-19 deaths were also more frequently associated with obesity in several countries [105,106,107]. In line with these results, a recent meta-analysis including 30 studies for a total of 45,650 participants demonstrated that BMI defined obesity was significantly associated with a higher risk for severe COVID-19 including, hospitalization (OR 2.36), intensive care unit (ICU) admission (OR 2.32) and invasive mechanical ventilation (OR 2.63), as well as for mortality (OR 1.49) [108]. Further, excessive visceral adiposity appeared to be significantly associated with severe COVID-19 outcomes [108,109].

The contribution of overweight/obesity to a more severe disease has biological and physiological plausibility. Obesity is characterized by abnormal secretion of several pro-inflammatory adipokines and cytokines from the adipose tissue, i.e., tumor necrosis factor-alfa, interleukin (IL)-1, IL-6, and monocyte chemoattractant protein, which are responsible for a low-grade, chronic pro-inflammatory state that may predispose to a greater COVID-19 severity [100,110,111]. Obesity may indirectly impact COVID-19 outcomes impairing lung function and respiratory system compliance to mechanical ventilation, thus placing these patients at high risk of severe illness and mortality [112]. In addition to the detrimental effects on lung function, obesity is a well-known risk factor for diabetes, hypertension and CVDs, all predictors of poor outcomes [93].

### 3.6. Cancer

Cancer patients are regarded as a highly vulnerable group due to weakened immune systems caused by both tumor growth and anticancer treatment [59,113,114]. In a nationwide cohort study on 2007 Chinese patients, Liang et al. [115] demonstrated that cancer patients had poorer outcomes from COVID-19. In fact, 39% of cancer patients experienced severe events, including ICU admission, need for invasive ventilation, or death, compared with only 8% of noncancer COVID-19 patients. Comparably, the analysis of data from the COVID-19 and Cancer Consortium (CCC19) cohort study on 928 cases also reported that 30 day-mortality and severe illness in COVID-19 cancer patients were significantly higher than the general population [116]. The most common malignancies in Kuderer et al. [116] were breast (21%) and prostate (16%) cancer; 43% had measurable cancer while 39% were on active cancer treatment.

Among 3000 early COVID-19 cases from Italy, a previous (5 years) history of cancer was reported by 20% of non-survivor patients [9]. Higher rates of death, ICU admission, need for invasive mechanical ventilation, the manifestation of at least one severe or critical symptom was reported by Dai et al. [117] on 105 cancer patients. In this population, the most frequent cancer type was lung cancer (20.95%), followed by gastrointestinal (12.38%), breast (10.48%), thyroid (10.48%) and hematological cancers (8.57%). Moreover, the highest frequency of severe complications was reported in patients with hematological, lung, or metastatic cancer (stage IV). Concerning the role of treatment, patients who received surgery or an anticancer treatment within 14 days before COVID-19 diagnosis had higher risks of having severe events compared to patients without cancer, while such increase was not evident in those receiving only radiotherapy [118,119]. Patients who underwent chemotherapy or surgery in the prior month had a significantly higher risk (75%) of clinically severe events than those who did not receive chemotherapy or surgery (43%) [115]. Similarly, another study of 205 COVID-19 cancer patients showed that receiving chemotherapy within four weeks before symptoms onset and male sex were risk factors for death [120].

### 3.7. Respiratory Diseases

The association between respiratory diseases and severe COVID-19 outcomes has not been definitively clarified. Concerning the prevalence of respiratory diseases among 13,184 patients with COVID-19, a recent metanalysis demonstrated that the chronic obstructive pulmonary disease (COPD) was the main respiratory disease documented [121], and only one study also reported pulmonary tuberculosis and asthma [122]. A significantly 4-fold higher odds of severe COVID-19 outcomes were determined in patients with underlying respiratory diseases, a result in line with those previously obtained by Yang et al. [123] in a smaller meta-analysis. A nearly four-to six-fold higher risk of developing severe COVID-19 was associated with the presence of COPD [121,124], although no significant association could be extrapolated for mortality association [124]. Few available studies have reported a high prevalence of asthma among COVID-19 patients, preventing definite conclusions [125,126].

### 3.8. Autoimmune Diseases

No definite conclusions can be extrapolated concerning the risk for COVID-19 infection and for more severe manifestations in patients with systemic autoimmune diseases in comparison with the general population with similar comorbidities [127,128,129,130,131]. A meta-analysis performed on 62 observational studies on autoimmune diseases that affected patients demonstrated that the prevalence of COVID-19 was 0.011 [128]. Among the seven case-controlled studies available, including psoriasis and rheumatic patients, the risk for COVID-19 was significantly higher than in controls (OR 2.19). This finding was also confirmed in an Italian series of systemic autoimmune diseases [129]. However, this was primarily attributed to the glucocorticoid use, while other therapies, including conventional (i.e., methotrexate, hydroxychloroquine, and sulfasalazine) or biological or target synthetic (i.e., infliximab, adalimumab, etanercept, tofacitinib) disease-modifying antirheumatic drugs (DMARDs) did not contribute to the risk [128]. No evidence for differences in hospitalization, ICU admission, mechanical/non-invasive ventilation, and death emerged from several investigations in comparison to the general population [128,132,133]. Only therapies with glucocorticoids, conventional DMARDs, combined conventional synthetic-biological/target synthetic DMARDs had a 2–3 times higher event rate of each clinical outcome when compared with biological-target synthetic DMARDs alone [128].

## 4. Occupational Risk Factors for SARS-CoV-2 Susceptibility

In workplace settings, the story is not simply one of understanding and grading individual risk factors, but also to put individual conditions in relation to the risk of contracting COVID-19 through work. In fact, in a complementary manner, occupational analysis can address workplace environmental risk of exposure to and acquisition of infection, while personal features can determine the impact of infection once acquired [134]. From an occupational health perspective, it is clear that susceptibility should be considered as the result of the complex interplay between individual and the job or task features. This regards several types of working conditions, like healthcare and social works, where biological risks are intrinsically related to the job activities, but also other occupational sectors for which the risk of infection is comparable to that of the general population.

Therefore, work has been recognized as a key determinant in the risk of infection [135]. Multiple outbreaks of COVID-19, in fact, have been reported in a variety of occupational fields, including food packaging and processing sectors, factories and manufacturing departments, and office settings [136]. As an ulterior confirmation, the analysis of the Italian compensation claims pointed out that the SARS-CoV-2 infection has been acquired at the workplace in a substantial number of cases (19.4% of the total amount) [137]. The probability of being in contact with infected people, the physical proximity to others during work activities, and the social aggregation connected to the job may function as key determinants in workplace SARS-CoV-2 transmission [135,138]. In the absence of control measures, a higher risk of infection may be experienced by workers involved in occupations in which it is difficult to maintain physical distancing from coworkers, customers, patients, as well as by employees performing work activities in indoor settings or with shared transport or accommodation [136].

In this scenario, methodological approaches have been developed to estimate the levels of the risk of infection associated with various worksites. These first explore the exposure probability due to specific work tasks, including where, how, and to what sources workers may be exposed to SARS-CoV-2. Such sources may be represented by the general public, customers, and coworkers; sick individuals, like in the case of healthcare workers, or those at particularly high risk of infection, but also non-occupational risk factors at home and in community settings depending on the local infection prevalence [139]. Additionally, the extent to which the job entails either close proximity to people who may carry the infection or contact with materials that may be contaminated by the virus, aggregation factors as well as the effectiveness of any measure to reduce transmission, i.e., barriers or personal protective equipment, as well as how the individual commutes to work should be deeply assessed [138].

According to all these aspects, job tasks may be categorized into different levels of exposure risk. From very high exposure risk jobs, like those in which healthcare or laboratory workers are exposed to aerosol-generating medical maneuvers or lab procedures requiring collecting or handling specimens from known or suspected COVID-19 patients, respectively, to high, medium and lower exposure risk jobs. This latter group includes jobs not requiring contact with people known or suspected to be infected with SARS-CoV-2 nor frequent close contact with the general public [139]. The importance of such classification relies on the corresponding preventive and protective protocols to be adopted to mitigate workplace transmission and the prompt identification and isolation of potentially infectious individuals within the company. According to the “hierarchy of controls”, such strategies may include (i) to remove or reduce the exposure at the source, e.g., through flexible work solutions, where possible, screening, testing, case investigating and contact tracing; (ii) to redesign the work environments with the adoption of barriers and protection elements to facilitate social distancing, and the implementation of areas for frequent hand washing and sanitizations; (iii) to support the adoption of organizational preventive measures in the workplace; (iv) to promote health and safety education of individual workers and the employment of personal protective equipment in order to minimize the exposure of receptors [140].

However, suitable risk assessment and management strategies in the workplace should also integrate susceptibility evaluation and careful considerations of specific conditions of the individuals, e.g., older age, presence of chronic medical conditions, including immunocompromising ones. These issues appear extremely important considering the global aging of the workforce [141,142] and the growing population of immunosuppressed people who enter or reenter the workforce [143]. Overall, this perspective allows moving of the risk assessment from a population-based to an individually personalized approach. Moreover, it, inevitably, underlines the central role of occupational physicians in integrating personal information with job or task features as a unique opportunity to achieve suitable management of susceptible individuals at work.

Risk assessment models should offer the possibility to quantify the vulnerabilities associated with demographic variables and comorbidities and their possible combined impact with occupational features in order to measure the equivalent increase in risk that may predict severe courses and case-fatality rate [1,144,145]. Age, gender, ethnicity and comorbidities are commonly included in occupational risk assessment tools, while measures of social circumstances are less frequently assessed [134]. This risk categorization should support individually tailored preventive and protective measures regarding workplace adaptations (e.g., unrestricted work or work-restricted to certain patient groups) or actions (e.g., staff redeployment or home working). However, caution should be paid in the application of such tools for workplace policymaking. Some limitations should be carefully considered, including the focus on the relative risk that is not able to assess whether the absolute risk is actually low, intermediate or high, and the possible lack of accuracy of such points score instruments where multiple risk factors co-exist that may provide an artificial classification of the risk. Finally, by addressing only “individual factors,” the scores do not include the interaction between these and the working environment or job task features. This points out the relevance of developing advanced models able to integrate all these aspects in order to have better representative risk assessment measures. Overall, clinical judgments, technical considerations, and prevailing advice from the government need to be carefully considered in managing occupational risks from COVID-19.

## 5. Discussion

The ongoing COVID-19 pandemic still requires a deeper understanding of the nature of the disease and its suitable treatment or preventive measures, as well as how to best safeguard public health in order to avoid the overwhelming of the health care system [146]. In this scenario, it also seems necessary to define appropriate strategies to identify and manage susceptible individuals with a higher risk of acquiring the disease and developing adverse outcomes. This means to translate data acquired on frail patients from clinical settings into a preventive perspective aimed to point out possible conditions of vulnerability that need specific attention and interventions in both public and occupational health contexts.

Concerning possible conditions of susceptibility to COVID-19, literature evidence reported age and sex as relevant demographic risk factors for more severe outcomes. Additionally, a list of underlying health conditions, particularly preexisting CVDs, diabetes mellitus, obesity and cancer were estimated to increase the risk for severe COVID-19. However, additional investigations are warranted to acquire a deeper knowledge with respect to the role of individual conditions or the association of personal and/or medical illnesses with respect to both the risk of acquiring COVID-19 and developing severe manifestations. In this regard, better-designed studies should be aimed to overcome possible limitations related to the self-reporting of comorbidities but also to the possible under-reporting of diseases due to the lack of awareness and/or diagnostic testing. A suitable duration of follow-up should also support the extrapolation of unbiased associations between comorbidities and clinical outcomes. Additionally, the limitations due to the heterogeneity of some categories of comorbidity should be overcome, i.e., chronic pulmonary disease aggregates various disorders that are characterized by a wide range of severity. Future investigations should allow evidence-based risk assessments for more specific subcategories of diseases or rarer comorbidities, also considering the detailed history of each condition and specific pathological characteristics. This may include disease onset and course, current or recent activity/flare-ups, past histories of hospital admissions, and medications (past, current or recent) used.

In the perspective of defining susceptibility conditions, a deep evaluation of possible consequences of SARS-CoV-2 infection should be included. In this view, the post-COVID-19 syndrome has been defined “as signs and symptoms that develop during or following an infection consistent with COVID-19, which continue for more than 12 weeks and are not explained by an alternative diagnosis. The condition usually presents as clusters of symptoms, often overlapping, which may change over time and can affect any system within the body” [147]. Notably, post-COVID-19 patients may exhibit extra-pulmonary manifestations, including fatigue, generalized pain, persisting high-temperature and psychiatric problems, neurological, cardiovascular, and musculoskeletal disorders, burdening their functional status [148,149]. Indeed, also physical, sensorial, cognitive, and emotional consequences in COVID-19 survivors should also deserve further investigation in order to better define their link with the previous COVID-19, considering the nonspecificity of most of these symptoms. Moreover, also their possible function as conditions of acquired vulnerability that may have implications for fitness for work also in the view of broader management of return to work in the face of COVID-19 health risks should be deeply addressed [150]. In this context, efforts for risk profiling based on a comprehensive assessment and testing markers of susceptibility in relation to outcomes, detection of early warning symptoms and atypical presentations among patients with multimorbidity should be strongly pursued. This may regard to define, i.e., biomarkers and immune function indicators able to predict how older adults’ immune responses help (or hinder) in fighting off the illness [151]. Finding possible biomarkers could be useful in identifying individuals at the highest risk for severe COVID-19 infection. Biomarkers may identify different risk management groups and yield important information on the mechanisms of severe disease. These may be associated with inflammation, endothelial function, mitochondria and apoptotic function, calcium homeostasis, fibrosis, neuromuscular function, sarcopenia, and bone/hormone metabolism and nutritional status [152,153].

As pointed out in previous paragraphs, on one hand, the work can impact a person’s risk to contract the infection; on the other hand, demographic and pathological factors can influence COVID-19 severity. Both aspects are critical in determining the risk of a poor outcome and need to be carefully addressed. Therefore, an occupational risk assessment should include the evaluation of the level of work-based risk to become infected and its mitigation, and personal characteristics that may determine the different impact of the infection. In this perspective, the valuable participation of occupational physicians in risk assessment and management processes appears essential to define plans to protect vulnerable workers, to advise on the fitness for work and to develop a suitable epidemiological surveillance system, all priority measures in effective anti-COVID-19 workplace strategies [1,135].

Additionally, it should be noted that the environmental and occupational risk, e.g., the prevalence of the disease at the community and hospital level, and the control strategies, e.g., measures to isolate infected patients, availability of personal protective equipment and vaccines, inevitably vary during the phases of the pandemic. This means that the risk assessment should not be viewed as a “one-off” process but as an iterative procedure to be updated when circumstances, whether environmental, occupational or individual, change. Overall, in actual conditions in which some uncertainties remain on several aspects, any risk assessment may be useful as a starting point for a discussion and the adoption of personalized preventive and protective plans [134]. To this aim, it seems important to underline that the complex interplay between the great variety of individual, physiological and pathological conditions and the huge spectrum of occupational realities requires a “case-by-case” approach to guide risk assessment and management measures. Any attempt to categorize personal or workplace features, in fact, can result in the failure of individually tailored preventive approaches that may result in an ineffective protection of susceptible workers.

Occupational health settings may represent appropriate scenarios for the early identification of vulnerable subjects. However, this mission finds its strategic importance once it is able to guide risk assessment and management procedures. This latter may include the systematic surveillance of work-related risk factors, collective preventive policies, the adoption of more stringent actions specifically focused on particular groups of the population (e.g., stricter measures of personal hygiene; implemented personal protective equipment; prioritized access to the ongoing vaccine campaign), and decisions on occupational placement of workers (e.g., remote working; changes in job tasks) [151]. Suitable management strategies can also regard the need to implement the general preventive measures characterized by worker/patient information on occupational risk mitigations and education to recognize signs and symptoms of COVID-19, as well as to follow strict respiratory and hand hygiene. Health promotion activities tailored to support healthy behaviors, lifestyle, emotional health and wellbeing in vulnerable workers should be developed. Regular physical exercise and the use of functional food, smoking cessation programming and the importance of appropriate management of chronic conditions should be stressed in specifically focused populations [154,155,156]. The use of remote monitoring, teleconsultation, telemedicine and online apps and resources may be innovative means to achieve suitable risk communication, maintaining strong messaging to promote compliance with key protective behaviors. These tools can also assure vulnerable subjects regular follow-up, and constant provision of health, behavioral and psychological support, which may help in applying specific recommendations for facing COVID-19 related risks in the workplace.

## 6. Conclusions

A suitable assessment of susceptibility conditions will provide guidance to develop effective surveillance regimens both through strict testing plans intended to reduce the asymptomatic spread and the rapid activation of contact tracing to reduce transmission and overall mitigate the impact on vulnerable individuals [157]. Additionally, a deeper knowledge of vulnerability may be useful to establish priorities in vaccination strategies [158] and to define policies for the correct use of public resources of insurance systems [138]. Overall, additional studies should be aimed to verify the impact that policy measures adopted to face the pandemic may have on vulnerable groups. Certainly, as the knowledge about COVID-19 risks further develops, risk assessments and management for workplace risk factors and individuals, particularly vulnerable subjects, will need to be reviewed. Concerted actions of general practitioners, hospital specialists, occupational physicians and all figures involved in the health and safety management in the workplace should be strongly encouraged to plan suitable preventive measures for vulnerable subjects [159].
